# Safety and Effectiveness of Muse Cell Transplantation in a Large-Animal Model of Hepatic Fibrosis

**DOI:** 10.1155/sci/6699571

**Published:** 2025-06-13

**Authors:** Taketo Nishina, Hiroaki Haga, Shohei Wakao, Keita Maki, Kei Mizuno, Tomohiro Katsumi, Kyoko Tomita Hoshikawa, Takafumi Saito, Masahiro Iseki, Michiaki Unno, Mari Dezawa, Yoshiyuki Ueno

**Affiliations:** ^1^Department of Gastroenterology, Yamagata University Faculty of Medicine, 2-2-2 Iidanishi, Yamagata 990-9585, Japan; ^2^Department of Stem Cell Biology and Histology, Tohoku University Graduate School of Medicine, 1-1 Seiryo, Sendai 980-8575, Japan; ^3^Department of Hepatobiliary Surgery, Tohoku University Graduate School of Medicine, 1-1 Seiryo, Sendai 980-8574, Japan

## Abstract

**Background:** In recent years, liver regeneration therapy using mesenchymal stem cells (MSC) has been investigated as an alternative therapy for end-stage liver diseases. Among these MSCs, multilineage-differentiating stress enduring (Muse) cells are reported to be effective in mouse models. The present study investigated the safety and effectiveness of Muse cell transplantation in large animal models of hepatic fibrosis.

**Methods:** Muse cells and MSC were prepared from bone marrow cells of male mini pigs (Göttingen strain). Recipients mini pigs (female Göttingen strain) were repeatedly administered with carbon tetrachloride (CCl_4_) intraperitoneally for 12 weeks to induce liver fibrosis. Thereafter, either Muse cells or MSCs were transplanted intravenously. After the cell transplantation, laboratory tests, vital signs, and liver histology were evaluated (Muse cell group (*n* = 6), MSC group (*n* = 6), and vehicle group (*n* = 7)).

**Results:** Liver fibrogenesis was successfully induced after 12 weeks of CCl_4_ administration. Engraftment of transplanted cells and differentiation into hepatocytes were confirmed in recipients' liver. In Muse cell group, significant increase of serum albumin (Alb) level was observed at 4 weeks compared to those of control groups (*p*  < 0.05). Hepatic proliferating cell nuclear antigen (PCNA) positive cells were significantly increased in the Muse cell group (*p*  < 0.05). Hepatic fibrogenesis at 12 weeks after transplantation were significantly improved in Muse cell group (*p*  < 0.05). Alpha-smooth muscle actin (α-SMA) immunostaining revealed significant decrease in liver from Muse cell transplanted recipients. No serious adverse effects were observed.

**Conclusions:** Muse cell transplantation was safe and effective in large animal models of hepatic fibrosis. The positive effects were observed in namely 4 weeks after transplantation. Since biochemical as well as histological improvements were demonstrated, future studies including establishing ideal administration protocol seem to be feasible as a preclinical study.

## 1. Introduction

End stage liver diseases are serious medical conditions which require liver transplantation as an only life-saving treatment [1]. However, due to the shortage of donations, liver transplantation is not applicable for every patient with end stage liver diseases [1, 2]. In most countries, the hepatitis virus related liver diseases are leading cause of liver cirrhosis [2–4] Even introduction of directly acting antivirals (DAAs) for hepatitis C virus related liver diseases, certain proportion of patients develop liver failure even after viral eradication [3–5]. Also, hepatitis B related liver cirrhosis is mostly managed with nucleot(s)id treatment, whereas some patients with advanced liver fibrosis reportedly deteriorates after intervention [6, 7]. Furthermore, there are increased number of patients with decompensated cirrhosis from several liver diseases such as alcoholic liver cirrhosis or nonalcoholic fatty liver diseases [8, 9]. Thus, it is essential to develop life-saving medical therapy other than liver transplantation [10].

Autologous bone marrow and stem cell transplantation has been clinically applied for liver generation, especially in Japan [11]. However, it is not widely used in a general clinical setting. In the field of regenerative medicine, induced pluripotent stem (iPS) cells prepared by genetic modification have been considered to be promising therapeutic options, and thus, several clinical trials using iPS cells has been conducted [12]. In the past, clinical trials with mesenchymal stem cell (MSC) have been applied for liver disease and improvements of hepatic reserves and hepatic fibrogenesis have been reported [13, 14]. However, recent clinical trials could not find the beneficial effects of MSC transplantation [15, 16]. Although other reports and studies suggested the potential beneficial effects of cellular transplantation as clinical applications, they are not conclusive [17–19]. Thus, development of alternative methods, especially using large animal model was warranted [20].

One of the candidates for cell source is multilineage-differentiating stress enduring (Muse) cells [21, 22]. Muse cells exist in one out of 3000 bone marrow cells and are also found in skin and fat [23]. In addition, Muse cells differentiate into cells of tridermic system (ectoderm, mesoderm, and endoderm). Muse cells are characterized by positivity of both the MSC marker, CD105, and the pluripotent stem cell marker, stage-specific embryonic antigen-3 (SSEA-3) [24]. Since Muse cells could be isolated using marker SSEA-3, and they have enough pluripotency without the transgene techniques [25]. In an in vivo study using Muse cells, unlike embryonic stem (ES) cells and iPS cells, there is no reported formation of teratoma; thus, the risk of developing tumor formation is considered low [26].

In a Muse cell transplantation experiment with a murine liver fibrogenesis model, recognition and accumulation to the damaged organ was observed [15]. As for hepatic regeneration, human Muse cells transplanted into mice model with 70% liver resection, Muse cells differentiated into not only hepatocytes but also bile duct epithelial cells, sinusoidal endothelial cells, and Kupffer cells [27]. However, the efficacy of Muse cell transplantation in large animals has not been investigated before clinical application to human end-stage liver diseases. Liver of mini pigs (Göttingen mini pig) has similar external appearance, volumes, and hematological/biochemical levels to human livers. Thus, we prepared a liver fibrosis model using carbon tetrachloride (CCl_4_) to mini pigs to induce hepatic fibrosis model and examined the therapeutic effect of Muse cells transplantation assessed by several indicators.

## 2. Materials and Methods

### 2.1. Induction of Liver Fibrosis Model Using Mini Pigs

Mini pigs (Göttingen breed, 12-month-old females and 12-month-old males) were purchased from Oriental Yeast Industry Company (Tokyo, Japan) and kept in the Animal Experimentation Center of Yamagata University. Animal experiments were handled strictly in accordance with the Law Concerning the Protection and Management of Animals. The animals were kept in a special cage at a room temperature of 20–24°C. The diet was MP-A ordinary diet (Oriental Yeast Co., Ltd.) and fed once a day at 400 g/day. All the animal experiments were approved by Yamagata University Animal Experiment Committee (Approval No. 28082).

To create the liver fibrosis model, the initial dose of CCl_4_ was administered as 0.12 or 0.15 mg/kg intraperitoneally to female mini pigs, referring to the dose in the mouse liver fibrosis model. Thereafter, 0.15 mg/kg of CCl_4_ was administered once a week for a total of 12 weeks. At this point, liver biopsy specimen was collected for histological evaluation by hematoxylin and eosin staining and Elastica– Masson staining, as well as blood samples for routine laboratory tests.

### 2.2. Isolation and Culture of Cells for Transplantation

Bone marrow fluid was collected from the femur of a male Göttingen mini pig and the bone marrow fluid was incubated in Dulbecco's modified eagle's medium with 10% fetal bovine serum and human fibroblast growth factor-2 (Miltenyi Biotec, Bergisch Gladbach, Germany; 1 ng/μl) was added to the cell culture medium at 37°C, 5% CO_2_. After 3 days, only adherent cells were further cultured under the identical conditions for 14 days. Cultured cells were separated using flow cytometry with anti-mouse SSEA-3 antibody (IgG2b, BioLegend, San Diego, CA, USA) [24, 25] and further labeled green fluorescent protein (GFP) by lentivirus (80% transduction rate) as identical to our previous reports [27 ]. Above established protocol for establishing Muse cells were reported previously [26]. The Muse cells obtained in this experiment were isolated using flow cytometry. Muse cells (SSEA-3 positive) isolated by flow cytometry were used for cell transplantation in the current study. Thus, we utilized different fraction obtained by positive selection of SSEA-3 positivity as a cell surface marker. This protocol had resulted in the purity that over 95% of the cells were Muse cells.

### 2.3. Cell Transplantation

Total of 19 female mini pigs were used as the recipients of cell transplantation after induction of liver fibrosis models. The frozen transplanted cells were rapidly thawed, centrifuged, and dissolved with 10 ml of saline, and 1 × 10^7^ of either Muse (*n* = 6) or MSC (*n* = 6) cells per animal were slowly administered intravenously through the ear vein. The control group for cell transplantation (vehicle group) received 10 ml of saline and was assigned as vehicle group (*n* = 7). The viabilities of transplanted cells in this study were 93.4% ± 2.1% for MSCs and 92.1% ± 1.9% for Muse cells, with no significant difference. For histological evaluation, liver biopsies were taken at the time of cell transplantation and weekly liver biopsies were performed. Venous blood samples were collected weekly after cellular transplantation for routine laboratory tests. At 4, 8, and 12 weeks after cellular transplantation, 1–2 animals from each group were sacrificed to evaluate systemic organ evaluation (autopsy). Some portions of organ tissue as well as blood samples were stocked in a deep freezer for molecular and immunohistochemical analysis.

### 2.4. Blood and Biochemical Tests

Biochemical analysis was performed using Fuji Drychem NX500 (Fuji Film, Tokyo, Japan) and a biochemical kit (for humans, Fuji Film). CoaguChek XS (Roche Diagnostics K.K, Basel, Switzerland) and CoaguChek XS PT Test (Roche Diagnostics K.K) were used to measure prothrombin activity (PT%) in whole blood. All these assays were performed directly after collecting blood samples.

### 2.5. Pathological Analysis and Immunostaining

Liver tissues were fixed in 10% buffered formalin for 6–12 h, paraffin-embedded, and thinly sliced to 3 μm thickness for hematoxylin–eosin staining, Elastica–Masson staining, and immunostaining. For immunostaining, the samples were deparaffinized and treated with autoclave for antigen activation at 121°C for 15 min. Mouse monoclonal antihuman α-smooth muscle actin (α-SMA) antibody (IgG*κ*, DAKO, Carpinteria, CA, USA) was used as primary antibody and reacted overnight at 4°C. Then, EnVision System-HRP Labeled polymer anti-mouse (IgG, DAKO) was reacted as the secondary antibody for 30 min at room temperature and chromogenic with diaminobenzidine (Musashi Chemical, Tokyo, Japan). α-SMA immunostaining was performed on pancreatic tissue and saline was used instead of primary antibody for negative control. In addition, a monoclonal proliferating cell nuclear antigen (PCNA) antibody (IgG2a, PC10, Nichirei Bioscience, Tokyo, Japan) was reacted overnight at 4°C after the similar antigen activation treatment, followed by a secondary. The reaction was followed by a reaction with Histofine Simple Stain MAX-PO (MULTI; IgG, Nichirei Bioscience, Tokyo, Japan) as a secondary antibody for 30 min at room temperature and color development with ImPACT NovaRED (SK-4805, Funakoshi Co., Ltd., Tokyo, Japan). For GFP–albumin (Alb) fluorescence double staining, the same antigen activation treatment was performed, followed by overnight reaction with chicken GFP antibody (IgG, abcam, Cambridge, UK) at 4°C, followed by reaction with donkey anti-chicken Alexa488 antibody (IgG, Jackson ImmunoReseach, West Grove, PA, USA) for 2 h. Then, rabbit anti-pig Alb antibody (IgG, abcam), reacted overnight at 4°C, followed by donkey anti-rabbit Alexa594 antibody (IgG, Jackson ImmunoReseach) for 2 h, and encapsulated using an encapsulating agent (Diamond antifade mountant with DAPI). GFP-labeled Muse cells and MSCs were used as GFP-positive controls. Saline was used instead of primary antibody for negative control. For quantification of liver fibrosis, liver tissue stained with Elastica–Masson stain was observed using an all-in-one fluorescence microscope BZ-X710 (filter conditions for fluorescence observation, absorption wavelengths: GFP green 525 nm, Alb red 630 nm, DAPI blue 460 nm; KEYENCE, Osaka, Japan), at the magnification ×200. The background area of 10 fields of view was measured and the percentage of the area of blue fibrosis regions was evaluated. In PCNA immunostaining, background hepatocyte nuclei and PCNA-positive cell nuclei were measured using the cell count on the BZ-X710 microscope (KEYENCE, Osaka, Japan). The labeling index (LI), the percentage of PCNA-positive cells, was evaluated automatically.

### 2.6. Statistical Analysis

For biochemical test data, measurements were expressed as mean ± standard error and the values were used for statistical analysis. For histological analysis, each quantified data was expressed as a proportion and the mean ± standard error; one-way ANOVA test was used for comparisons among the three groups, followed by the multiple comparison test (Turkey method) for comparisons between the two groups and multiple comparison test (Turkey method) for time series within the same group. In all tests, *p* < 0.05 was considered significantly different.

## 3. Result

### 3.1. Induction of Liver Injury and Liver Fibrosis in Mini Pig Models

**Figure 1:**
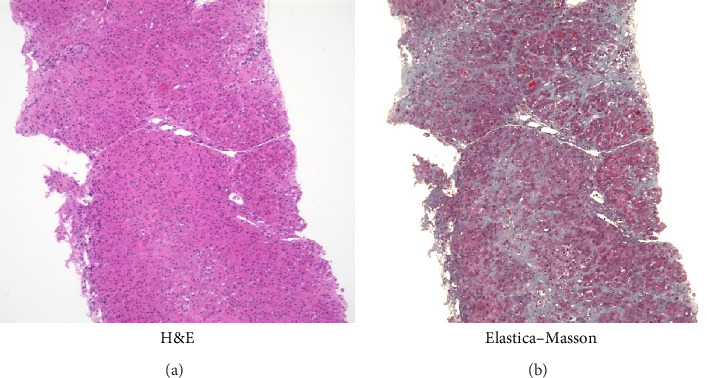
(a) Liver tissue specimens after 12 weeks of carbon tetrachloride treatment (before cell transplantation) demonstrate the infiltration of inflammatory cells in the liver tissue. (b) Hepatic fibrosis was observed in the portal vein area and the fibrosis extended into the hepatic lobule, similar to human cirrhosis (Elastica–Masson stain, ×100).

The initial dose of 0.15 mg/kg of CCl_4_ killed four of the eight animals (50% lethality after 1 day) and the initial dose of 0.12 mg/kg killed one of the eight animals (12.5% lethality after 1 day; Table 1). After an initial dose of 0.12 mg/kg, most of the animals survived the subsequent weekly doses of 0.15 mg/kg. Therefore, as a model protocol for liver fibrosis, CCl_4_ was administered at 0.12 mg/kg for the first time, followed by 0.15 mg/kg/week for 12 weeks. The body weight and blood and biochemical findings before (at the beginning of the experiment) and after 12 weeks of administration of CCl_4_ were similar to those observed in humans (Table 2). Liver biopsy specimen after 12 weeks of repeated intraperitoneal administration of CCl_4_ showed inflammatory cell infiltration and fibrosis formation in the liver parenchyma, similar to that of human cirrhosis (Figure 1).

### 3.2. Safety of Muse Cell Transplantation

All the recipients of cellular transplantation as well as vehicle treatment had no adverse effects after transplantation. Body temperature, body weight, food intake, and vital signs (blood pressure) had no significant changes during the observation periods (data not shown).

### 3.3. Evaluation of Pig Muse Cells and MSCs After Transplantation

**Figure 2:**
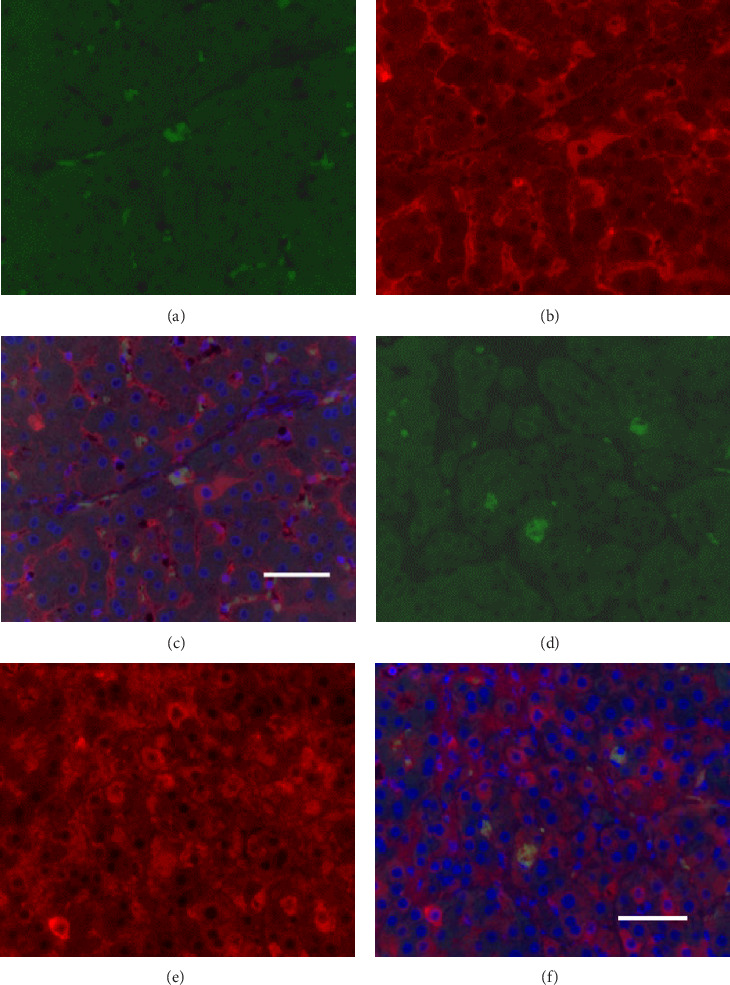
Immunofluorescence of GFP (green; a, d), Albumin (red; b, e) and GFP–Alb double immunostaining (yellow; c, f) in liver tissue specimens 4 weeks after cell transplantation. GFP–Alb double immunostaining was found in liver tissue in the Muse cell group (*n* = 6) and MSC group (*n* = 6; c, f; ×400). (a) Muse group (GFP–positive cells). (b) Muse group (GFP–Alb co–positive cells). (c) Muse group (Alb–positive cells).

In the liver tissues of the Muse and MSC groups, 1.6 ± 0.2 and 1.2 ± 0.2 GFP (green) and Alb (red) co-positive cells (yellow) were observed in average of one 400x field of view, respectively, confirming the engraftment and differentiation into hepatocytes in both groups (Figure 2).

### 3.4. Evaluation of Hepatic Reserve and Fibrosis After Cell Transplantation

**Figure 3:**
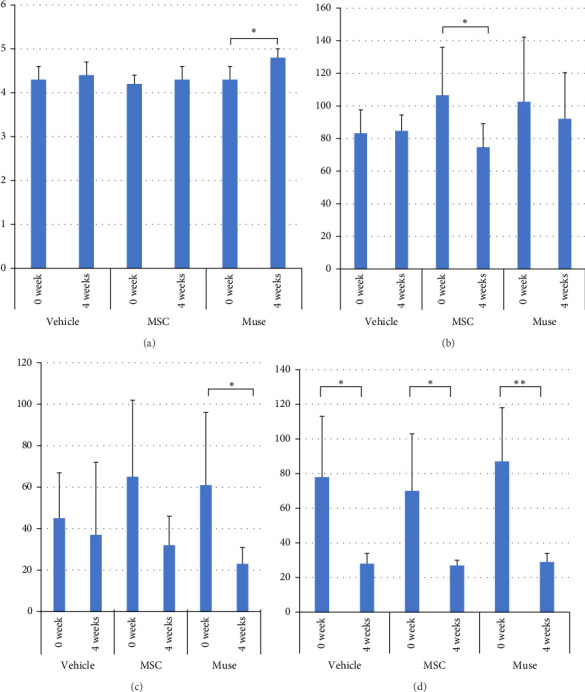
Biochemical tests before cell transplantation (0 week) and 4 weeks after cell transplantation (4 week): There were no significant differences between the Muse cell group (*n* = 6), MSC group (*n* = 6), and vehicle group (*n* = 7) in any of the parameters before cell transplantation (0 week). Serum albumin (a) in the Muse cell group, NH_3_ (b), and ALT (c) in the MSC group and AST (d) in all groups were significantly improved after 4 weeks of transplantation compared to before transplantation. (a) Serum albumin levels after cellular transplantation. (b) NH_3_ levels after cellular transplantation. (c) AST levels after cellular transplantation. (d) ALT levels after cellular transplantation. *⁣*^*∗*^*p* < 0.05; *⁣*^*∗∗*^*p* < 0.01.

There were no significant differences among the three groups about the backgrounds at the time of cell transplantation. Regarding the laboratory parameters (T. Bil, AST, ALT, Alb, ammonia (NH_3_)), white blood cell count, hemoglobin, and PT%, there were significant differences among the three groups (Table 3). At 4 weeks after transplantation, there was a significant improvement in serum Alb levels in the Muse cell group among the three groups (4.35 ± 0.31 g/dl before transplantation and 4.66 ± 0.19 g/dl at 4 weeks, *p* < 0.05, Figure 3a). Similarly, NH_3_ levels in the MSC group showed significant decrease compared to before transplantation (106.5 ± 29.4 μg/dl before transplantation and 74.6 ± 14.6 μg/dl at 4 weeks, *p* < 0.05, Figure 3b). Regarding AST values, AST levels in the MSC group showed significant improvement (65.6 ± 38.9 IU/L before transplantation and 23.5 ± 7.2 IU/L at 4 weeks, *p* < 0.05, Figure 3c). ALT values significantly improved in each group 4 weeks after transplantation (26.6 ± 6.2 U/l in the Vehicle group, 27.3 ± 2.5 U/l in the MSC group, and 29.0 ± 4.6 U/l in the Muse group, *p* < 0.05, Figure 3d).

**Figure 4:**
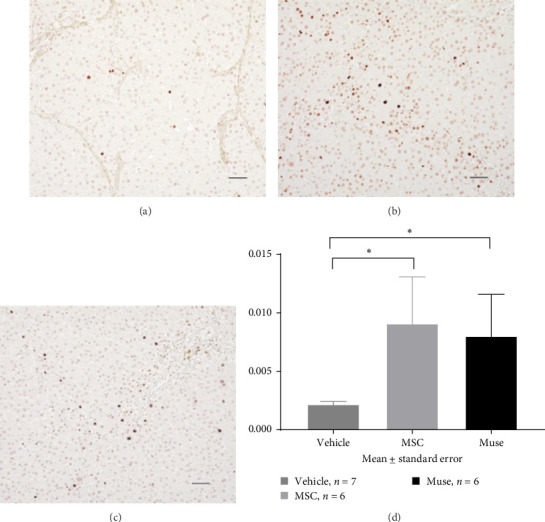
PCNA immunostaining in liver tissue specimens 4 weeks after cell transplantation: Representative examples of PCNA immunostaining in the (a) Vehicle group (*n* = 7), (b) MSC group (*n* = 6), and (c) Muse cell group (*n* = 6) are shown (×200). (d) Labeling index (LI) of PCNA–positive cells shows a significant increase in PCNA labeling rate in the MSC and Muse cell groups compared to the Vehicle group (4d). *⁣*^*∗*^*p* < 0.05.

**Figure 5:**
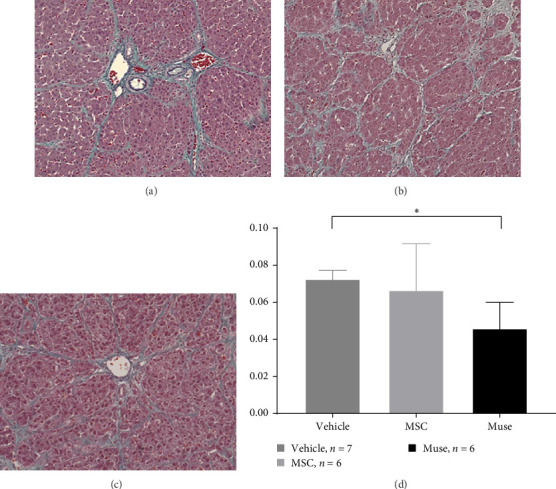
Evaluation of liver fibrosis after 12 weeks of cell transplantation (Elastica–Masson staining): Representative examples of staining in the (a) vehicle group (*n* = 7), (b) MSC group (*n* = 6), and (c) Muse cell group (*n* = 6) are shown (×200). (d) Percentage of positive area of Elastica–Masson staining versus background area, demonstrating significant improvement of liver fibrosis in the Muse group compared to the vehicle group (d). Statistical tests between the three groups were performed by multiple comparison test (Turkey method). *⁣*^*∗*^*p* < 0.05.

**Figure 6:**
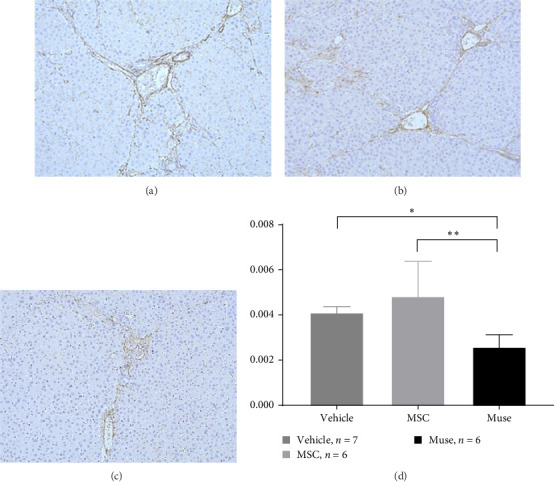
Assessment of hepatic stellate cell activation (α-SMA immunostaining) after 12 weeks of cell transplantation: Representative examples of immunostaining in the (a) vehicle group (*n* = 7), (b) MSC group (*n* = 6), and (c) Muse cell group (*n* = 6) are shown (×200). (d) Percentage of positive area of α-SMA immunostaining versus background area. Significant reduction of α-SMA positive cells (improvement of liver fibrosis) was observed in the Muse group compared to the vehicle and MSC groups (d). Statistical tests among the three groups were performed by multiple comparison test (Turkey method). *⁣*^*∗*^*p* < 0.05; *⁣*^*∗∗*^*p* < 0.01.

PCNA immunostaining showed a significant increase in positive cells in the Muse and MSC groups compared to the Vehicle group 4 weeks after transplantation (0.0029 ± 0.0002 in the Vehicle group vs 0.0091 ± 0.0040 in the MSC group, *p* < 0.05:0.0029 ± 0.0002 in the Vehicle group vs Muse group 0.0080 ± 0.0036, *p* < 0.05, Figure 4). Evaluation of liver fibrosis by Elastica Masson staining showed a significant reduction in the Muse group compared to the Vehicle group, with significant fibrosis improvement (Vehicle group 0.073 ± 0.005 vs MSC group 0.067 ± 0.025, *p* < 0.05, Figure 5). *α*-SMA immunostaining, significantly decreased *α*-SMA positive area in the Muse group compared to the Vehicle and MSC groups (0.0041 ± 0.0002 in the Vehicle group vs 0.0048 ± 0.0015 in the MSC group, 0.0041 ± 0.0002 in the Vehicle group vs 0.0025 ± 0.0004 in the Muse group, *p* < 0.05, Figure 6).

## 4. Discussion

In this study, we successfully established the mini pig model of hepatic fibrosis by repeated intraperitoneal administration of CCl4. Also, the transplantation of Muse cells into the mini-pig was safe as like MSC transplantation. Furthermore, cellular transplantation in hepatic fibrosis model resulted in an increase in hepatic reserve capacity and cell proliferation activity, as well as improvement of hepatic fibrosis, demonstrating the usefulness of Muse cell transplantation for the first time in large animal models.

Recently, ES cells, iPS cells, and MSCs have been candidates for transplantation cell sources as a liver regeneration therapy, and basic research has been conducted on each of them [28–30]. However, the risk of tumorigenesis is a concern with utilization of ES cells and iPS cells. Muse cells are considered to be pluripotent-like stem cells with low risk of tumorigenesis after transplantation because, unlike ES and iPS cells, they do not require fertilized eggs or exogenous gene transfer. In the area of liver diseases, improvement of hepatic fibrosis has been reported by transplanting Muse cells into a mouse model of hepatic fibrosis [27]. However, these beneficial effects were not evaluated in large animals, thus basic research using large animals is needed for future clinical application to humans.

In this study, we first established a hepatic fibrosis model in mini pigs for the first time. To do this, we fine-tuned the dosage of CCl_4_ since the ordinal dosage used in rodents were fatal for female mini pigs since half of those pigs died of hepatic failure within 48 h at this dosage. This may be due to the high sensitivity of mini pigs to carbon tetrachloride which has not been reported elsewhere. The authors reduced this fatality by reducing the initial dose of CCl_4_ from 0.15 to 0.12 mg/kg, and increased the maintenance dose as 0.15 mg/kg to maintain hepatic fibrosis assuring the safety of surviving animals. In addition, even CCl_4_ administration was discontinued at cellular transplantation, we confirmed that hepatic fibrosis was maintained in the liver tissue harvested after 12 weeks of discontinuation (Figures 5 and 6). Furthermore, the macroscopic appearance, hepatic pathology, and laboratory tests were similar to those of humans (Table 2), suggesting that our mini pig model is an appropriate large animal model for future clinical application to humans.

Four weeks after cellular transplantation of allogeneic Muse cells into a mini-pig liver fibrosis model, GFP and Alb co-expressing cells were observed in the liver tissue, confirming the differentiation of Muse cells into recipient's hepatocytes (Figure 3). Compared to the reported mouse model (Muse cell engraftment rate: ~6%) [27], the number of GFP-Alb co-positive cells in this study was 1.6 ± 0.2 cells/view (400x) in the Muse group and 1.2 ± 0.2 cells/view (400x) in the MSC group, indicating that fewer cells were engrafted in our model. The reasons for this may include the possibility that the transplanted Muse cells were eliminated by the immune response because the previous mouse model used an immunodeficient model, whereas our study used a normal mini pig model, thus, most of transplanted cells in our model could be eliminated during differentiation into hepatocyte. This issue will be addressed by autologous cellular transplantation model in future studies.

PCNA staining showed a significant increase of PCNA-positive cells in the Muse and MSC group compared to the Vehicle group at 4 weeks after cell transplantation (Figure 4), indicating that cell proliferation activity was enhanced in both cellular transplantation groups. Blood biochemical tests showed a significant increase in Alb levels, and a decreasing tendency in NH_3_ levels in the Muse group at 4 weeks after cell transplantation. These results implicated that Muse cell transplantation can improve liver function and regeneration in the liver.

In our study, significant improvement of hepatic fibrosis was observed in the Muse and MSC transplanted groups compared to the vehicle group at 12 weeks after transplantation (Figures 5 and 6). The mechanism involved in this improvement may be derived from possible humoral factors secreted from Muse cells. In a previous study in a mouse model, it was confirmed that MMP-9 was significantly increased in Muse cell transplantation compared to MSCs [27], thus, the similar mechanism may be involved in the present study. Our recent model using surgical hepatic resection model similarly demonstrated the involvement of MMP-9 which is highly expressed in Muse cells [27]. Moreover, in that study, we found mini pig Muse cells could spontaneously differentiate into hepatocytes [28]. In addition, Wakao et al. [31] demonstrated differentiation of human Muse cells into hepatocytes in vitro. Conversely, MSCs are shown to also exhibit phagocytic activity; however, their differentiation potential remains limited, and although they differentiate into cells of the same mesodermal lineage (etc., chondrocytes and adipocytes), they do not differentiate beyond the germline [31]. In this previous report, they showed that Muse cells phagocytose dead differentiated cells (in this case, dead hepatocytes) and recycle the natural pardon factors that were functioning in the hepatocytes, resulting in efficient differentiation in a short period of time [31]. In the brain, it has also been shown in vivo that Muse cells phagocytose the dead neurons and differentiate into neurons [31]. Apart from this mechanism, hepatic stellate cells are known to play the central role in hepatic fibrosis and apoptosis and inactivation of stellate cells as well as antifibrotic chemokines such as CX3CR1 [32, 33] could be evaluated in mini pigs model in future different studies. Moreover, more recent sophisticated techniques such as applying single cell analysis will help our understanding of the precise mechanisms of in vivo cellular mechanisms [34].

Until now, liver fibrosis models have mainly been created in small animals such as mice and rats, although it is unrealistic to apply the dosage of small animals to human applications. Thus, establishing large animal models seems to be feasible to develop novel cell transplantation therapy before human clinical trials. Recently, mini pig hepatectomy model has been reported to apply Muse cell transplantation of surgical hepatic failure model [27]. With this context, we believe that the results of our study strongly support the usefulness of Muse cell transplantation in human diseases such as end-stage liver cirrhosis.

One limitation with this study is that the number of studies is still small. The number of studies was limited due to the expensive cost of mini pigs resulting from limited number of pigs available in Japan for medical experiments. Another limitation is the large individual differences among the mini pigs. In this study, although laboratory studies at the time of liver injury showed no significant differences among the groups, there was a large variation in regard of body weight, which may affect the individual response to the cellular transplantation. We believe this issue could be addressed in future studies. Last, low availability of the molecular and immunohistochemical agents made it difficult to apply modern molecular or immunological approach to analyze possible mechanisms observed in our study. This is one of the fundamental and intrinsic disadvantages of using mini pigs.

In conclusion, although further investigation with more large numbers of animals is needed, current study demonstrates that Muse cell transplantation seems to be safe and beneficial to ameliorate hepatic fibrosis in large animal models. Future studies seem to be feasible to confirm our observations to bridge possible human clinical trials.

## Figures and Tables

**Table 1:** Initial dose of carbon tetrachloride and its lethality in mini pigs after 1 day of CCl_4_ administration.

CCl_4_ dosage (mg/kg)	Total number of individuals	Number of deaths	Number of survivors	Lethality (%)
0.12	8	1	7	12.5
0.15	8	4	4	50.0

**Table 2:** Laboratory tests before and 12 weeks after CCl_4_ administration.

Checklist	Before carbon tetrachloride administration (mean ± standard error) (*n* = 19)	12 weeks after carbon tetrachloride administration (mean ± standard error) (*n* = 19)
Weight (kg)	24.5 ± 4.9	23.1 ± 4.4
T.Bil (mg/dL)	0.11 ± 0.04	0.14 ± 0.06
AST (U/L)	23.0 ± 11.5	57.1 ± 30.6
ALT (U/L)	29.0 ± 4.7	82.8 ± 37.1
γ-GTP (U/L)	43.8 ± 6.5	67.7 ± 19.5
ALP (U/L)	199.2 ± 47.9	189.8 ± 54.7
TP (g/dL)	6.73 ± 0.36	6.77 ± 0.48
Alb (g/dL)	4.71 ± 0.23	4.25 ± 0.48
NH_3_ (μg/dL)	105.8 ± 17.6	95.1 ± 27.8
WBC (/μL)	6721 ± 1,031	6879 ± 1,382
Hb (g/dL)	11.4 ± 1.0	10.5 ± 2.4
Plt (×10/L)^3^μ	431.1 ± 99.6	613.5 ± 135.2
PT (%)	68.4 ± 8.3	73.3 ± 9.8

Abbreviations: γ-GTP, gamma-glutamyl transpeptidase; Alb, albumin; ALP, alkaline phosphatase; ALT, alanine aminotransferase; AST, aspartate aminotransferase; CCl_4_, carbon tetrachloride; Hb, hemoglobin; NH_3_, ammonium; Plt, platelet counts; PT, prothrombin time; T.Bil, total bilirubin; TP, total protein; WBC, white blood cell.

**Table 3:** Laboratory tests of vehicle, MSC, and Muse cell groups at the time of cellular transplantation.

Items	Vehicle (*n* = 7) (mean ± standard error)	MSC (*n* = 6) (mean ± standard error)	Muse (*n* = 6) (mean ± standard error)	*p* Value
Age (month)	14.1 ± 1.1	15.6 ± 1.3	15.3 ± 2.0	N.S.
T.Bil (mg/dL)	0.14 ± 0.05	0.16 ± 0.10	0.13 ± 0.05	N.S.
AST (U/L)	45.0 ± 22.4	65.6 ± 38.9	61.5 ± 35.7	N.S.
ALT (U/L)	78.5 ± 35.3	62.5 ± 33.3	87.8 ± 31.6	N.S.
Alb (g/dL)	4.30 ± 0.67	4.25 ± 0.24	4.35 ± 0.31	N.S.
NH_3_ (μg/dL)	83.2 ± 14.4	106.5 ± 29.4	102.5 ± 39.7	N.S.
WBC (/μL)	7084 ± 1158	6373 ± 1777	6778 ± 1438	N.S.
Hb (g/dL)	10.1 ± 3.8	10.4 ± 0.73	11.8 ± 1.69	N.S.
PT (%)	74.4 ± 14.8	68.6 ± 5.75	75.0 ± 6.89	N.S.

## Data Availability

The data that support the findings of this study are available from the corresponding author upon reasonable request.

## Nomenclature

CCl_4_: Carbon tetrachloride
T.Bil: Total bilirubin
AST: Aspartate aminotransferase
ALT: Alanine aminotransferase
γ-GTP: Gamma-glutamyl transpeptidase
ALP: Alkaline phosphatase
TP: Total protein
Alb: Albumin
NH_3_: Ammonium
WBC: White blood cell
Hb: Hemoglobin
Plt: Platelet counts
PT: Prothrombin time
Muse cells: Multilineage-differentiating stress enduring cells
MSC: Mesenchymal stem cells.
